# Sphingosine-1 phosphate promotes thymic atrophy during sepsis progression

**DOI:** 10.1186/cc14054

**Published:** 2014-12-03

**Authors:** L Kuchler, LK Sha, AK Giegerich, T Knape, A Weigert, B Brüne, A von Knethen

**Affiliations:** 1 Faculty of Medicine, Institute of Biochemistry I - Pathobiochemistry, Goethe-University Frankfurt, Germany; 2 Project Group Translational Medicine & Pharmacology TMP, Fraunhofer Institute for Molecular Biology and Applied Ecology IME, Frankfurt, Germany

## Introduction

T-cell depletion is a marker of the hypo-inflammatory phase of sepsis [1,2]. Systemic T-cell loss could be caused i.a. by thymus involution during sepsis, thus contributing to immune paralysis [3]. Usually, thymic T-cell egress is mediated by a sphingosine-1-phosphate (S1P) gradient. That is, T cells leave the thymus towards an increased S1P-level in the periphery (blood/lymph) [4]. A disruption of this S1P gradient blocks T-cell emigration. S1P is produced by sphingosine kinases 1 and 2 (SPHK1 and SPHK2), which are ubiquitously expressed [5]. Apoptotic cells are known to release S1P [6]. We suggest that apoptotic events in the thymus during sepsis increase S1P levels, thereby disrupting the gradient and preventing egress of T cells from the thymus.

## Methods

We used a murine polymicrobial sepsis model to analyze thymus involution. Sepsis is induced in wildtype mice, SPHK1^-/-^, or SPHK2^-/- ^mice by cecal ligation and puncture (CLP). Thymus involution was determined by analyzing the T-cell amount of single-positive mature (CD4^+^CD8^- ^vs. CD4^-^CD8^+^), double-positive late immature (CD4^+^/CD8^+^), and double-negative early immature cells by FACS analysis. T cells from SPHK1^-/- ^or SPHK2^-/- ^mice, which produce less S1P, and wildtype mice treated with a SPHK1 inhibitor were used to characterize whether T cells in these mice have a higher rate of emigration compared to control mice. Thymic and serum S1P levels were quantified by LC-MS/MS. Apoptosis was determined by annexin V FACS staining. SPHK mRNA levels were determined by qPCR.

## Results

The thymus of septic mice showed more CD3-positive cells but a decreased number of CD4/CD8 double-positive T cells, pointing to a thymic retention of single-positive mature T cells. In line with our assumption, CLP causes apoptosis of thymocytes and increases SPHK1 mRNA expression. Concomitantly S1P levels are increased in the thymus and consequently decreased in serum following CLP. The knockout of the S1P producing sphingosine-kinases SPHK1 or SPHK2 and the pharmacological inhibition of SPHK1 restores T-cell egress as indicated by an increase of double-positive immature T cells compared to wildtype mice.

## Conclusion

Our data suggest that inhibition of SPHK1-mediated S1P generation during sepsis restores thymic T-cell egress, which might improve septic outcome. Therefore, understanding mechanisms of thymus involution during sepsis may help to reconstitute thymic function, finally improving immune reactions in the hypoinflammatory phase of sepsis.
